# Nox2 dependent redox-regulation of microglial response to amyloid-β stimulation and microgliosis in aging

**DOI:** 10.1038/s41598-020-58422-8

**Published:** 2020-01-31

**Authors:** Li Geng, Lampson M. Fan, Fangfei Liu, Colin Smith, Jian -Mei Li

**Affiliations:** 1 School of Biological Sciences, University of Reading, Reading, UK; 2 Faculty of Health and Medical Sciences, University of Surrey, Guildford, UK; 3 Faculty of Cardiovascular Medicine, University of Oxford, Oxford, UK; 4 Centre for Clinical Brain Sciences, University of Edinburgh, Edinburgh, UK

**Keywords:** Biochemistry, Neuroscience, Pathogenesis

## Abstract

Microglia express constitutively a Nox2 enzyme that is involved in neuroinflammation by the generation of reactive oxygen species (ROS). Amyloid β (Aβ) plays a crucial role in Alzheimer’s disease. However, the mechanism of Aβ-induced microglial dysfunction and redox-regulation of microgliosis in aging remains unclear. In this study, we examined Nox2-derived ROS in mediating microglial response to Aβ peptide 1–42 (Aβ_42_) stimulation *in vitro*, in aging-associated microgliosis *in vivo* and in post-mortem human samples. Compared to controls, Aβ_42_ markedly induced BV2 cell ROS production, Nox2 expression, p47^phox^ and ERK1/2 phosphorylation, cell proliferation and IL-1β secretion. All these changes could be inhibited to the control levels in the presence of Nox2 inhibitor or superoxide scavenger. Compared to young (3–4 months) controls, midbrain tissues from wild-type aging mice (20–22 months) had significantly higher levels of Nox2-derived ROS production, Aβ deposition, microgliosis and IL-1β production. However, these aging-related changes were reduced or absent in Nox2 knockout aging mice. Clinical significance of aging-associated Nox2 activation, microgliosis and IL-1β production was investigated using post-mortem midbrain tissues of humans at young (25–38 years) and old age (61–85 years). In conclusion, Nox2-dependent redox-signalling is crucial in microglial response to Aβ_42_ stimulation and in aging-associated microgliosis and brain inflammation.

## Introduction

Microglial cells are the resident immune cells of the central nervous system (CNS) and play an important role in neuroinflammatory responses and the development of aging-related neurodegenerative diseases^1,2^. Microglial cells express constitutively an O_2_^.−^ generating Nox2 (also called gp91^phox^) containing NADPH oxidase^3^. When the cells face challenges from pathophysiological insults, the microglial NADPH oxidase is activated and produces large amount of ROS as a part of the immuno-defence responses to protect the CNS and in the meantime causes inflammatory damage to brain tissue^4^. Microglial cells can also respond to intrinsic metabolic neurotoxic by-products or protein aggregates, such as amyloid-β (Aβ) peptide, and become activated. This process can be long-lived, self-perpetuating and play a key role in driving the progression of age-related neurodegenerative diseases such as Alzheimer’s disease (AD)^5^.

One of the pathological characteristics of AD is the extracellular deposition of the Aβ peptide into amyloid fibril plaques^6,7^. Aβ (1–42) fragment (Aβ_42_) is the dominant Aβ species found in amyloid plaques of AD patients^8^. Microglia display proinflammatory phenotype in contact with Aβ to phagocytose and digest Aβ for its clearance and to build up a barrier to prevent neurotoxic effects of Aβ plaques^9,10^. Increased NADPH oxidase activity was found in the brain tissues of animal models of neurodegenerative diseases^11–14^ and in post-mortem brains of AD patients^15,16^. Inhibition or knockout of Nox2 significantly reduced aging-associated metabolic disorders and brain oxidative stress and preserved locomotor function in aging mice^17,18^. Microglia derived from Nox2 knockout (Nox2KO) mice had no ROS response to neurotoxin stimulation^19^. However, the role of Nox2-derived ROS and redox-signalling in mediating microglial response to Aβ stimulation and microgliosis in normal aging remains unclear.

In this study we investigated the role of Nox2-derived ROS and the key redox-signalling pathways in mediating microglial response to Aβ_42_ stimulation *in vitro*. The crucial role of Nox2 in normal aging-associated oxidative stress, microgliosis, ERK1/2 activation, protein tyrosine nitridation and inflammatory cytokine (IL-1β) production was examined using midbrain tissues (containing hippocampus and VTA regions) of littermates of age-matched wild-type (WT) and Nox2 knockout (Nox2KO) mice at young (3–4 months) and old (20–22 months) age. Aging-associated Nox2 activation, microgliosis and IL-1β production were further confirmed using human post-mortem midbrain samples at young (25–38 years) and old age (61–85 years). Our data provide novel information of a crucial role of Nox2 and redox-signalling in mediating microglial response to Aβ_42_ stimulation and aging-associated microgliosis and brain inflammation.

## Results

### Effects of Aβ42 on BV2 microglial cell ROS production and cell vitality

**Figure 1:**
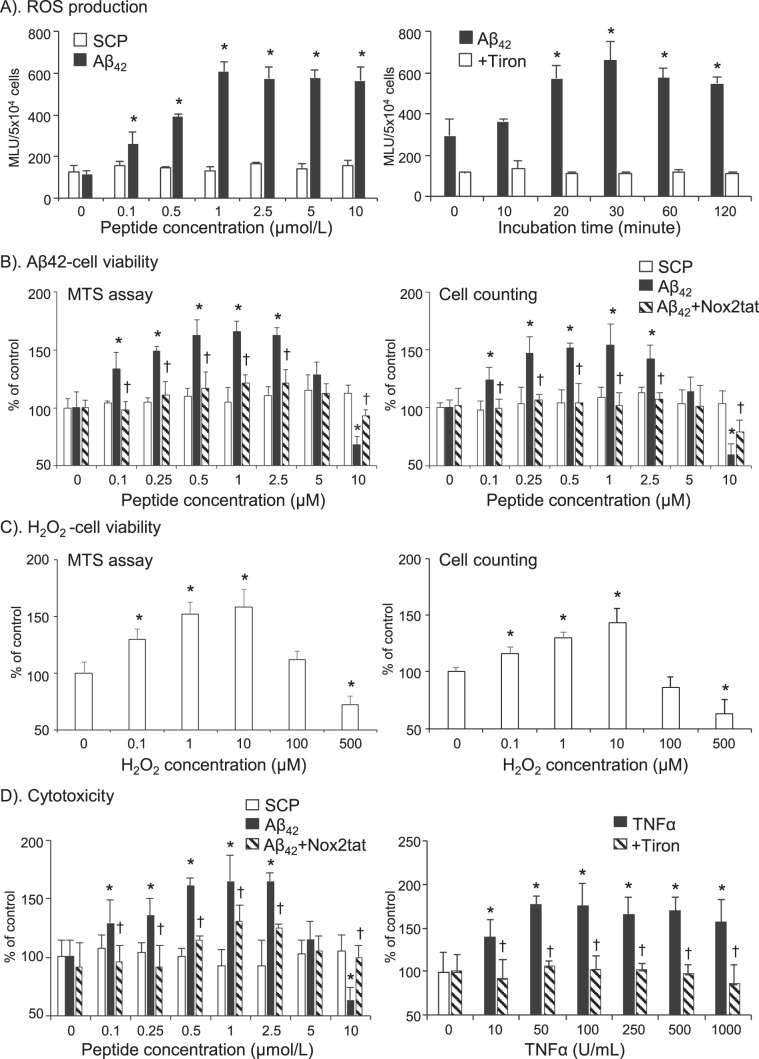
The effects of Aβ_42_ on BV2 cell ROS production and cell viability. (**A**) ROS production detected by lucigenin-chemiluminescence. Left panel: Aβ_42_ dose response. Right panel: Aβ_42_ stimulation time course. Tiron was used to confirm the detection of O_2_^.−^. (**B**) Effects of Aβ_42_ on cell viability detected by MTS assay (left panel) or by trypan blue exclusion (right panel). (**C**) Effects of H_2_O_2_ on cell viability detected by MTS assay (left panel) or by trypan blue exclusion (right panel). (**D**) Cell cytotoxicity detected by MTT assay. SCR: scrambled control peptide. Nox2tat was used to inhibit Nox2-derived ROS production. n = 5 independent cell cultures. *P < 0.05 for indicated values versus values at 0 point under the same treatment. ^†^P < 0.05 for indicated values versus Aβ_42_ (or TNFα) values without inhibitor in the same treatment group.

BV2 cells had been well characterized and used as a substitute for primary cells in many previous studies of mouse microglial functions^20–22^. Therefore, we examined the effects of Aβ_42_ on BV2 cell O_2_^.−^ production using lucigenin chemiluminescence in cell suspension (Fig. 1A). Compared to SCP, Aβ_42_ (24 h) increased markedly the O_2_^.−^ production by BV2 cell in a dose-dependent manner starting at 0.1 µmol/L, peaking at 1 µmol/L and then stayed at a plateau form (Fig. 1A, left panel). Time course showed that BV2 cells started to increase ROS production after 10 min of Aβ_42_ stimulation, peaked at ~30 min and remained high up to 2 h of Aβ_42_ stimulation (Fig. 1A, right panel). The effects of Aβ_42_ on BV2 cell viability was examined by CellTiter 96 AQueous One Solution Cell Proliferation Assay (MTS) (Fig. 1B, left panel) and trypan blue exclusion (Fig. 1B, right panel). Aβ_42_ (24 h) at concentrations ≤ 5 µmol promoted BV2 cell proliferation and caused cell death at higher concentrations (Fig. 1B). Aβ_42_ induced BV2 cell proliferation was Nox2-derived ROS dependent and was abolished in the presence of a specific Nox2 inhibitor (Nox2tat). The role of ROS in mediating microglial proliferation was further investigated using H_2_O_2_ (without Aβ_42_) (Fig. 1C). H_2_O_2_ (24 h incubation) used below 100 µM induced BV2 cell proliferation and showed cytotoxicity above 100 µM.

We also used the traditional MTT assay to examine BV2 cell cytotoxicity in response to Aβ_42_ (Fig. 1D, left panel) and to TNFα (Fig. 1D, right panel). Aβ_42_ showed  cytotoxicity to BV2 cell at doses above 5 µmol (Fig. 1D, left panel). However, different from Aβ_42_, TNFα showed no cytotoxicity to BV2 cells even at a very high dose of 1000 Unit/ml (Fig. 1D, right panel). TNFα-induced BV2 cell proliferation was ROS dependent and could be abolished in the presence of tiron, an O_2_^.−^ scavenger.

**Figure 2:**
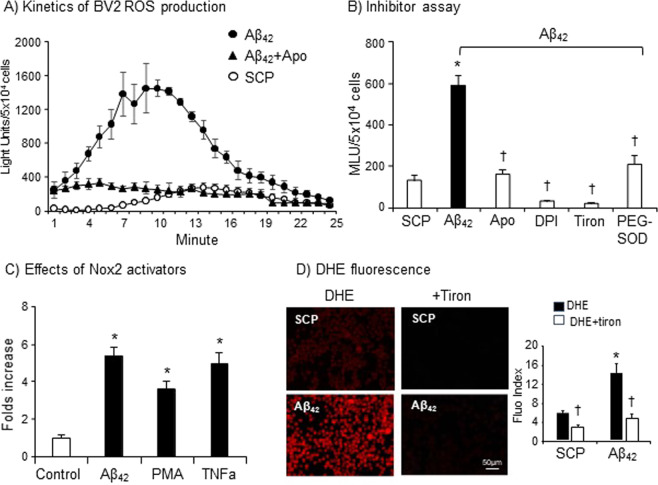
Effects of Nox2 inhibitors or activators on BV2 cell O_2_^.−^ production detected by lucigenin-chemiluminescence (**A–C**) and DHE fluorescence (**D**). (**A**) Real-time recording of BV2 cell O_2_^.−^ production. (**B**) Effect of Nox2 inhibitors, apocynin (Apo) and DPI on Aβ_42_-induced O_2_^.−^ production. Tiron and PGE-SOD were used to scavenge O_2_^.−^. (**C**) Comparison of the effects of Aβ_42_ (1 µM), PMA (100 ng/ml) and TNFα (100 U/ml) on BV2 cell O_2_^.−^ production. (**D**) *In situ* ROS production by adherend BV2 cells detected by DHE fluorescence. n = 5 independent cell culture experiments. *P < 0.05 for indicated values versus SCP values (**A,B,D**) or control values (**C**). ^†^P < 0.05 for indicated values versus Aβ_42_ values (**B**) or values without ROS scavenger (**D**) in the same treatment group.

The real-time production of O_2_^.−^ (detected every min for 25 min) by living BV2 cells in response to Aβ_42_ challenge (bolus dose, 1 µM) was further examined (Fig. 2A). Aβ_42_ increased the O_2_^.−^ production by BV2 cells progressively with the highest levels at 8–13 minutes of stimulation. Aβ_42_ induced increase in the O_2_^.−^ production by BV2 cells was completely inhibited by an NADPH oxidase inhibitor, apocynin (20 µmol/L). Compared to control cells stimulated with SCP, BV2 cells under Aβ_42_ stimulation increased ~6-folds of ROS production, which could be significantly inhibited by a Nox2 inhibitor (apocynin) or by a flavoprotein inhibitor (DPI) and was abolished in the presence of tiron (an O_2_^.−^ scavenger) or PEG-SOD (a superoxidase dismutase mimic) (Fig. 2B). Aβ_42_ (1 µM) was able to induce BV2 cells O_2_^.−^ production to a greater extent than PMA (100 ng/ml) and was equivalent to TNFα (100 U/ml) (Fig. 2C). Aβ_42-_induced ROS production by adherent BV2 cell was further confirmed by *in situ* DHE fluorescence (Fig. 2D).

### Aβ_42_ induced Nox2 expression, MAPK activation and Il-1β secretion by BV2 cells

**Figure 3:**
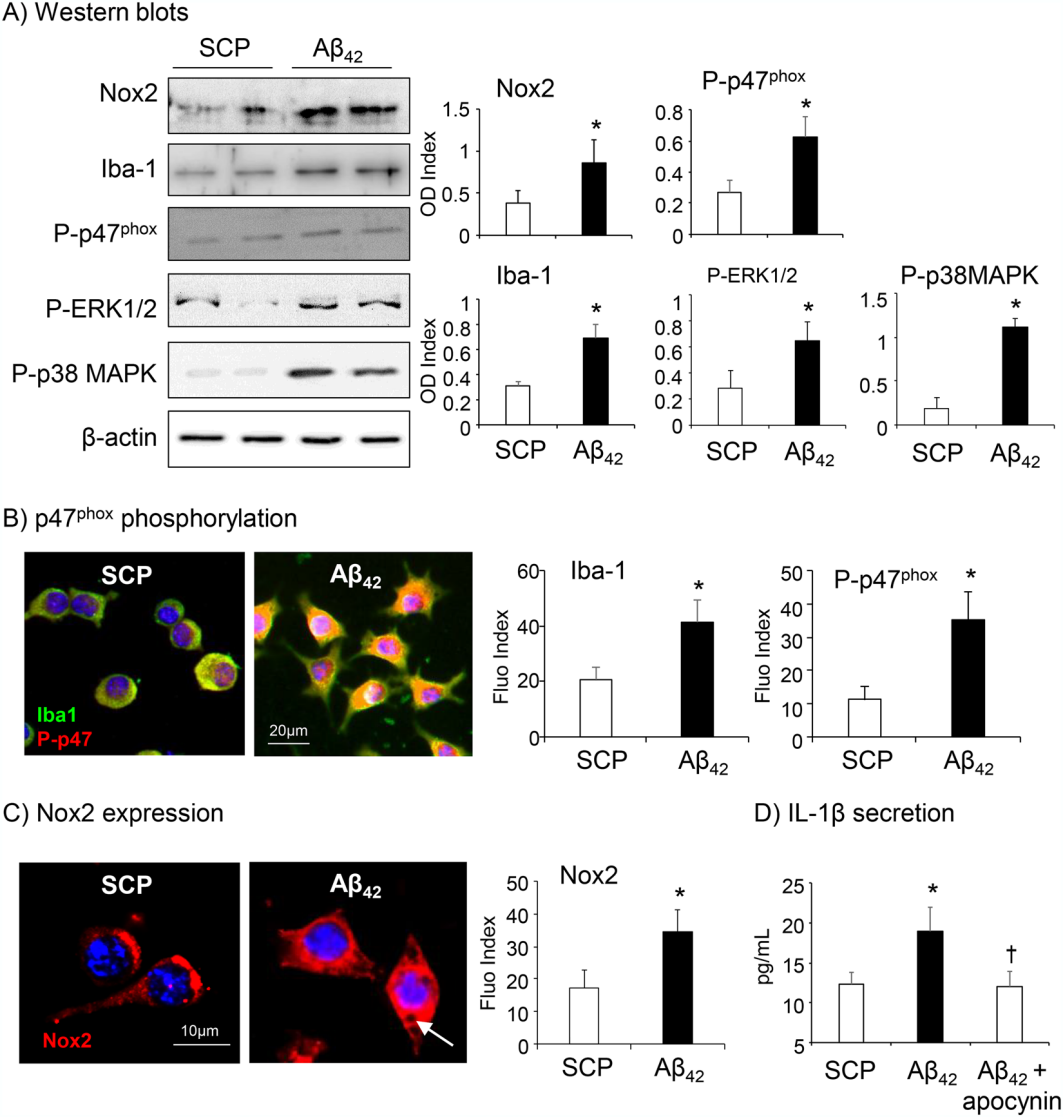
Aβ_42_-induced Iba-1 and Nox2 expression, the activation of stress-signalling pathways and IL-1β secretion by BV2 cells. (**A**) Western blots. Optical densities (ODs) of protein bands were quantified and normalized to β-actin (loading control) detected in the same sample. (**B**) p47^phox^ phosphorylation (red) was detected using a phosphorylation specific antibody against p47^phox^ (Ser359) and double stained with antibody against Iba-1 (green) by immunofluorescence. (**C**) Nox2 expression (red) detected by immunofluorescence. Nuclei were labelled by DAPI (blue) to visualise the cells. Fluorescence intensities were quantified, and expressed as index against controls without primary antibody. (**D**) IL-1β detected in the culture media by ELISA. n = 5 independent cell cultures. *P < 0.05 for indicated values versus SCP values. ^†^P < 0.05 for indicated values versus Aβ_42_ values.

BV2 cell Nox2 expression and the activation of redox-signalling pathways in response to Aβ_42_ stimulation were examined firstly by Western blots (Fig. 3A). Compared to SCP stimulated control cells, BV2 cells increased significantly the Nox2 protein expression in response to Aβ_42_ stimulation (24 h), and this was accompanied with increases in p47^phox^ phosphorylation (a key step in Nox2 activation), in expression of microglial ionized calcium binding adaptor molecule 1 (Iba-1) and the activation of stress signalling pathways, i.e. ERK1/2 and p38MAPK. Aβ_42_-induced subcellular expression of Iba-1 (green colour) and p47^phox^ phosphorylation (red colour) were further demonstrated by immunofluorescence staining (Fig. 3B). The yellow colour indicated the overlapping of Iba-1 and phos-p47^phox^ in Aβ_42_ stimulated microglial cells predominantly around the perinuclear and plasma membrane regions. Aβ_42_ -induced BV2 cell Nox2 expression was also examined by immunofluorescence (Fig. 3C). Accompanied with increased Nox2 expression, Aβ_42-_stimulated BV2 cells displayed visible phagocytic granules in the cytosol.

The effect of Aβ_42_ (24 h) on BV2 cell IL-1β secretion was examined by ELISA (Fig. 3D). In comparison to SCP stimulated cells, Aβ_42_ increased significantly the levels of IL-1β detected in the culture media of BV2 cells, which could be inhibited down to the control level by apocynin, a Nox2 inhibitor, suggesting a regulatory role of Nox2 in microglial function.

### Aging-associated Aβ deposition, microgliosis and Nox2 activation in WT and Nox2KO midbrain tissues

**Figure 4:**
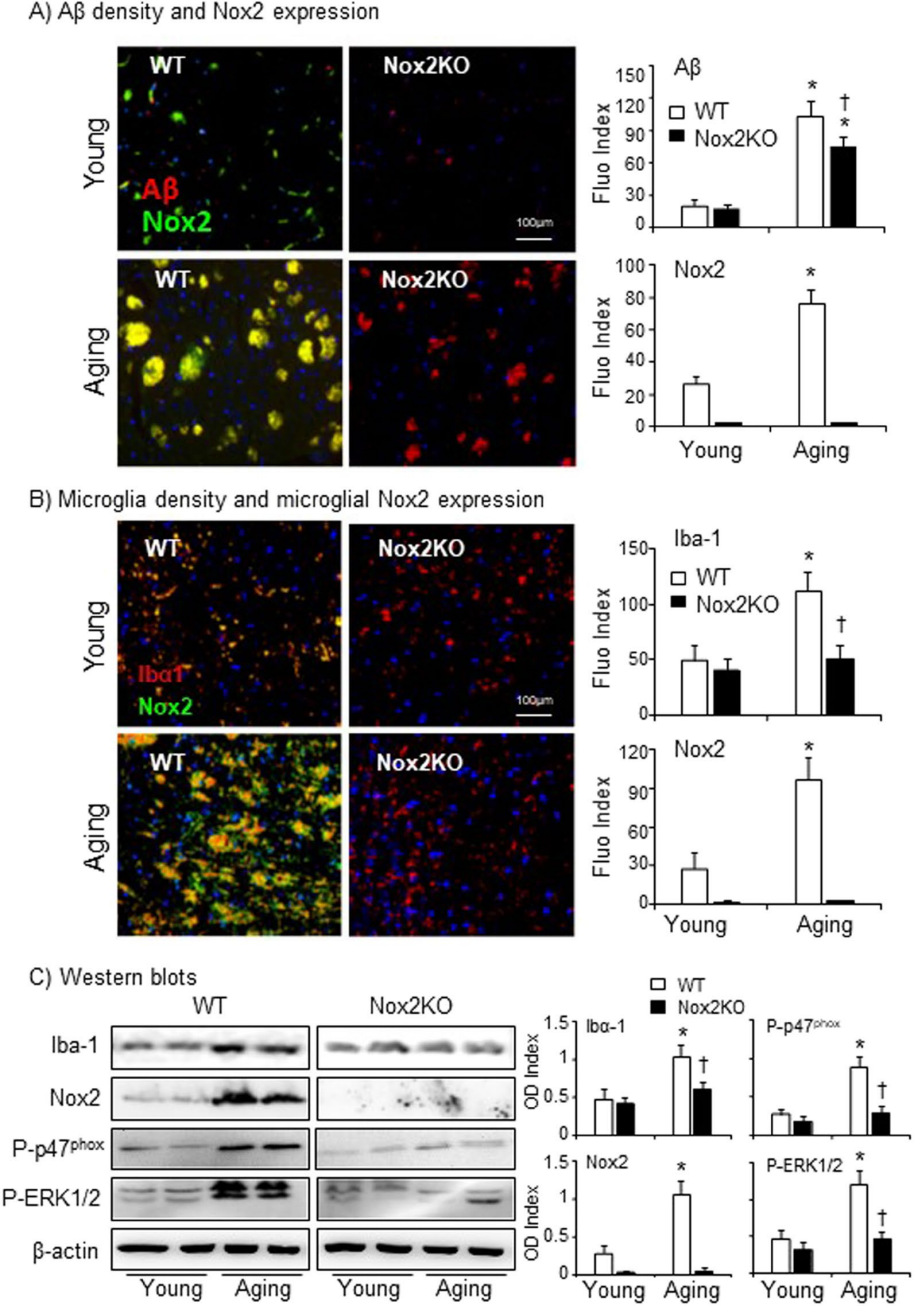
Aging-associated Aβ deposition and microglia proliferation in the midbrain tissues of WT and Nox2KO mice. (**A**) Immunofluorescence detection of Aβ deposition (red) and Nox2 expressions (green). (**B**) Immunofluorescence detection of microglia (labelled with Iba-1, red) and Nox2 (green). Fluorescence intensities were quantified, and expressed as index against control slides without primary antibody. Nuclei were labelled with DAPI (blue) to visualize the cells. (**C**) Western blot. Optical densities (ODs) of protein bands were quantified and normalized to β-actin detected in the same samples. n = 9 mice/per group. *P < 0.05 for indicated values versus young values of the same genetic group. ^†^P < 0.05 for indicated values versus WT values of the same age group.

Aβ aggregates had been found in aged C57BL/6 mouse brains, which was suggested to be a model to study pathogenesis of normal aging-associated Aβ plaque formation^23^. In order to explore the role of Nox2 and ROS in mediating Aβ induced microgliosis and inflammation in aging, we used the midbrain tissue sections of WT and Nox2KO mice of the same strain at young (3–4 m) and older age (20–22 m) to examine aging-associated Aβ deposition and Nox2 expression by double immunofluorescence (Fig. 4). Compared to their respective young controls, both WT and Nox2KO aging brains showed Aβ deposition and plaque formation (red colour). However, aging Nox2KO brains had significantly less Aβ deposition in comparison to Aging WT brains (Fig. 4A). WT aging brains had remarkably high levels of Nox2 expression (green colour) and a significant proportion of Nox2 was overlapped with the Aβ plaques (yellow colour) indicating the infiltration of Nox2-positive cells. We then examined microglial density and microglial Nox2 expression. Microglia were labelled by Iba-1 (a microglial marker, red) and Nox2 was labelled by FITC (green) (Fig. 4B). In comparison with WT young controls, we found significant increases in microglial density and Nox2 expression in WT aging brains. The superposed yellow colour indicated microglial Nox2 expression. Although the microglial density was also increased in aging Nox2 brains, the levels were much less than what were found in WT aging brains (Fig. 4B). Increased expressions of Iba-1and Nox2 and increased phosphorylation of p47^phox^ and ERK1/2 in the aging WT (not in aging Nox2KO) midbrain tissues were further confirmed by Western blots (Fig. 4C).

### Knockout Nox2 reduced brain ROS production, inhibited stress signalling, protein tyrosine nitration and IL-1β generation in aging

**Figure 5:**
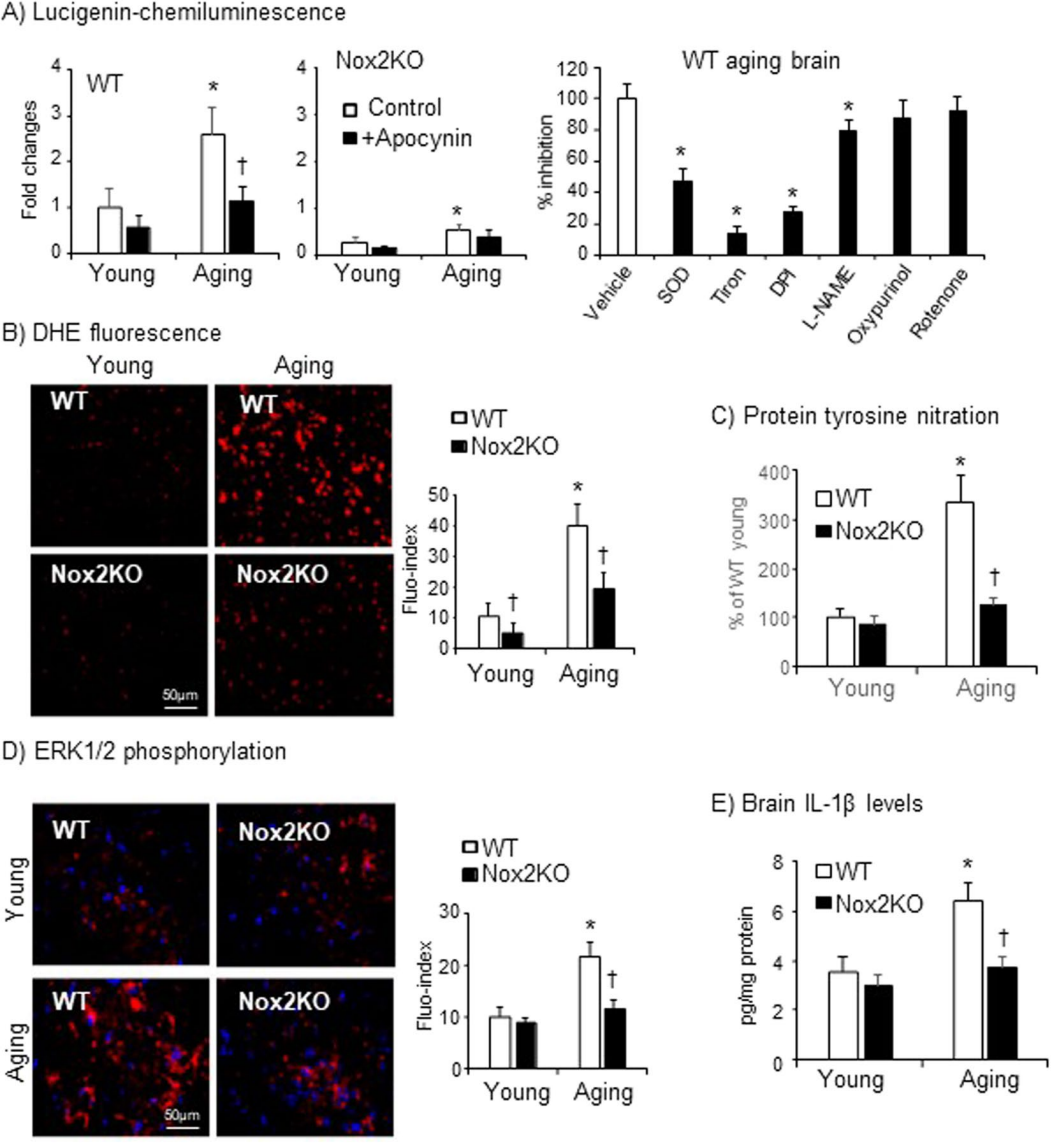
Aging-associated increase in ROS production, stress signalling activation and IL-1β production in the midbrain tissue of WT and Nox2KO mice. (**A**) O_2_^.−^ production detected by lucigenin-chemiluminescence using brain homogenates (left panels) and the effects of different enzyme inhibitors on WT aging brain ROS production (right panel). (**B**) *In situ* ROS production by midbrain sections detected using DHE fluorescence. (**C**) Protein tyrosine nitration detected using antibody against 3-NT by immunofluorescence on midbrain sections. 3-NT fluorescence intensity were quantified and expressed as % of WT young controls (expressed as 100%). (**D**) Aging associated ERK1/2 phosphorylation (red colour) detected using phos-ERK1/2 specific antibodies. (**E**) Levels of IL-1β detected in the midbrain tissue homogenates by ELISA. n = 9 mice/per group. *P < 0.05 for indicated values versus young values of the same genetic group, or values without inhibitor (A, right panel). ^†^P < 0.05 for indicated values versus WT values of the same age group.

The levels of brain O_2_^.−^ production were examined by lucigenin-chemiluminescence using midbrain tissue homogenates of WT and Nox2KO mice at young and old age (Fig. 5A). Compared to WT young controls, WT aging brains had significantly higher levels of O_2_^.−^ production, which could be reduced to the young control levels by adding apocynin (a Nox2 inhibitor), or DPI (a flavoprotein inhibitor) or L-NAME (an eNOS inhibitor), but not by rotenone (mitochondria complex 1 enzyme inhibitor) or oxypurinol (xanthine oxidase inhibitor) (Fig. 5A, right panel). In contrast, there were very low levels of O_2_^.−^ production by the midbrain tissues of Nox2KO mice at both young and old age. Aging-associated increase in ROS production in the aging WT brains was further examined by *in situ* DHE fluorescence using midbrain sections (Fig. 5B). Although the levels of ROS production in Nox2KO aging brains were also increased in comparison to Nox2KO young brains, the levels of increase were much lower than the levels detected in WT aging brains (Fig. 5B).

Superoxide reacts rapidly with NO to form peroxynitrite, a potent electrophile that forms stable 3-nitrotyrosine (3-NTyr) adducts of proteins that can be detected by immunofluorescence^24^. We detected the levels of protein tyrosine nitration using antibodies against 3-nitrotyrosin (3-NT) on midbrain sections (Fig. 5C). We found that accompanied with increased Nox2-derived O_2_^.−^ production, there were marked increases in the levels of protein nitration detected in the midbrains of aging WT mice but not in aging Nox2KO midbrains (Fig. 5C). Along with increased oxidative stress and protein nitration, there were increases in ERK1/2 activation (Fig. 5D) and in the levels of IL-1β production (Fig. 5E) detected in the WT aging brains. However, these aging-associated changes were significantly reduced in the absence of Nox2.

### Studies of human post-mortem midbrain tissues at young and old ages

**Figure 6:**
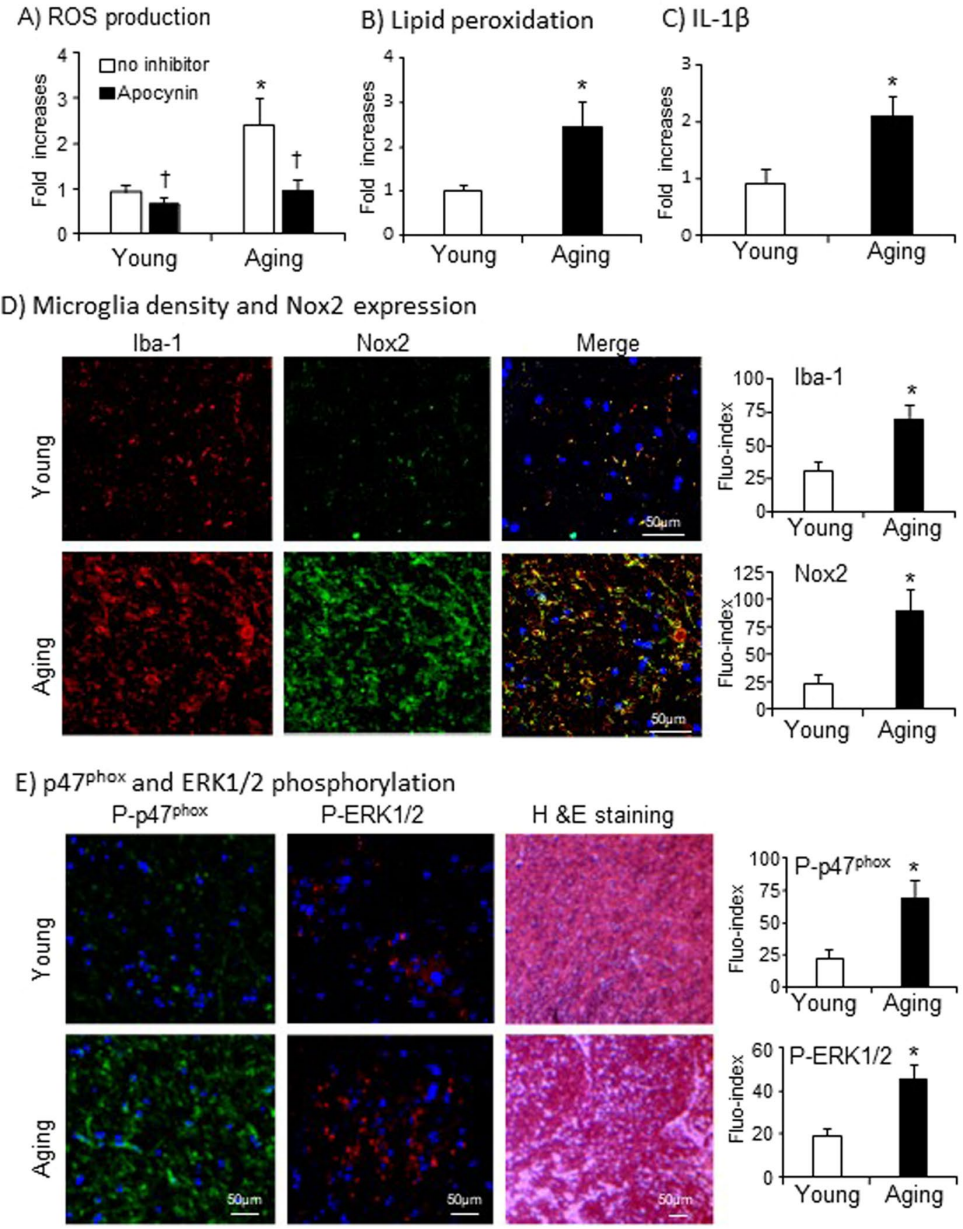
Aging-associated Nox2 activation, microgliosis and IL-1β production in post-mortem human midbrain tissues at young (25–38 years); and old age (61–85 years). (**A**) O_2_^.−^ production detected by lucigenin-chemiluminescence. (**B**) Lipid peroxidation detected by MDA assay. (**C**) Brain IL-1β levels detected by ELISA. Data were presented as fold changes against the young control values without inhibitor (expressed as 1). (**D**) Immunofluorescence detection of microglia density (labelled by Iba-1, red) and Nox2 expression (green). The yellow colour in merged images indicated the microglial expression of Nox2. (**E**) Immunofluorescence detection of p47^phox^ (green) and ERK1/2 (red) phosphorylation. Single channel fluorescence intensities were quantified. Young n = 7 and aging n = 8. *P < 0.05 for indicated values versus young values. ^†^P < 0.05 for indicated values versus values without inhibitor in the same age group.

The clinical significance of Nox2 activation in aging-associated microgliosis and inflammation was examined using post-mortem midbrain tissues (including the hippocampus and the VTA) of human adults at young (25–38 years old) and old age (61–85 years old) without diagnosed neurodegenerative diseases. Tissues were obtained from the UK Medical Research Council (MRC) tissue bank. Compared to young controls, aged human midbrain tissue had high levels of O_2_^.−^ production, which could be inhibited down to the young control levels in the presence of a Nox2 inhibitor, apocynin (Fig. 6A). Increased ROS production by human aging brains was further confirmed by increased lipid peroxidation detected using MDA assay (Fig. 6B). Along with increased ROS production, there were significant increases in brain production of inflammatory cytokine, IL-1β (Fig. 6C); in microglial density (labelled by Iba-1, red) and Nox2 expression (green) (Fig. 6D). The yellow color in the merged images indicated microglial Nox2 expression (Fig. 6D). The aging brains had remarkable levels of p47^phox^ phosphorylation (labelled green) and activation of stress signaling pathway, ERK1/2 (labelled in red) (Fig. 6E). The representative H&E staining showed visible white-matters in the midbrain sections of aging individuals in comparison to the sections of young adults (Fig. 6E).

## Discussion

Microglial dysfunction and increased ROS production by Nox2 enzyme are hallmarks of age-related neurodegenerative diseases^2,4^. Aβ_42_ is the dominant peptide found in AD plaques^8^. Microglia have been found adherent to Aβ plaques and to phagocytose Aβ in post-mortem brains of AD patients^9,10^. The current study using series of *in vitro* cytological and biochemistry assays, plus experiments using midbrain samples of age-matched littermates of WT and Nox2KO mice at young (3–4 m) and aging (20–22 m) and post-modem human midbrain tissues at young (25–38 years) and old age (61–85 years) provides evidences for the first time that: (1) Nox2-dependemnt ROS and redox-signalling regulated microglial responses to Aβ_42_ stimulation; (2) Knockout or inhibition of Nox2 protects aging brains from oxidative stress, microgliosis and IL-1β secretion.

Different from pathogen-induced oxidative burst described for neutrophils or blood monocytes^25^, we found that Aβ_42_ -induced BV2 microglia cell ROS production is a progressive reaction that needed around 10 min to reach the peak and lasts for hours. Along with increased ROS production, microglial cell proliferated in response to Aβ_42_ stimulation, which was inhibited in the presence of either Nox2 inhibitor or O_2_^.−^ scavenger. TNFα had been shown to induce microglial proliferation through its action on NADPH oxidase and ROS production^26,27^. It was suggested that Aβ-induced microglial proliferation was via the release of TNFα. In the current study, we found that both Aβ_42_ and TNFα were able to induce BV2 microglial proliferation. However, different from TNFα that is produced and released by microglia into the local extracellular environment, Aβ is phagocytosed by microglial cells and is cytotoxic at higher doses and could eventually kill microglial cells. Although BV-2 had been extensively characterized and used widely as a substitute for primary microglia in many experimental settings^20^, limitation should be considered when interpreting quantitative data obtained from BV2 cells. However, in the current study, the Nox2 activation and redox-regulation of microgliosis and IL-1 production in aging were confirmed by *in vivo* model of WT and Nox2KO mice and by using human brain samples at young and old age.

Previously, Aβ aggregates had been found in the brain tissues of aged WT C57BL/6 mice, and was suggested to be a model to study Aβ plaque formation in normal aging^23^. In addition, WT mice increased brain Aβ concentration when subjected to repeated mechanical stress or inflammation^28^. Adding to the existing literature, we showed in this study that Aβ deposition could be detected in the midbrain tissues of aging C57BL/6 mice using antibodies against Aβ by immunofluorescence specifically. Although knockout of Nox2 could not stop Aβ accumulation in the aging brain, the levels of Aβ found in aging Nox2KO midbrains were significantly less than in aging WT controls. Under the breeding condition without infection, we did not find any difference in life span between aged WT versus Nox2KO mice. In addition, Nox2KO aging brains had markedly less ROS production along with reduced levels of protein tyrosine nitration, p47^phox^ and ERK1/2 phosphorylation, microgliosis and IL-1β production.

Increased inflammatory cytokine production by activated microglia in the CNS is an indicator of neuroinflammation^29,30^. Interleukin-1 (IL-1) is one of the major inflammatory cytokines produced by microglia in response to Aβ stimulation^29^. An important finding from the current study is Nox2-derived ROS regulation of microglial IL-1β production. Inhibition of Nox2 by apocynin reduced BV2 cell IL-1β secretion in response to Aβ_42_ stimulation_,_ and knockout of Nox2 completely abolished aging-associated IL-1β production in the mid-brain tissue. The clinical significance of Nox2-derived ROS in aging associated microglial dysfunction was further demonstrated using post-mortem midbrain tissues of young (25–38 years old) and elderly (61–85 years old) adults without diagnosed neurodegenerative diseases. We showed that along with an increased brain ROS production, there were markedly up-regulations of microglial density, microglial Nox2 expression, phosphorylation of p47^phox^ and ERK1/2, and IL-1β production in the aging human midbrain tissues. Limitation should be considered when detecting lipid peroxidation by MDA in post-mortem human brain tissues due to the non-specificity of the assay or other possible issues after brain death^31^. However, in the current study, complementary technique of lucigenin-chemiluminescence with or without Nox2 inhibitor was also applied to measure brain ROS production.

In summary, we have presented in this study, by using several complementary approaches (*in vitro* cell culture, aging model of WT and Nox2KO mice, and human post-mortem brain tissues of young and elderly adults), an in-depth insight into the mechanisms of Nox2-dependent redox-regulation of microglial response to Aβ_42_ stimulation and a crucial role of Nox2-derived ROS in ageing-associated oxidative stress, microgliosis and Il-1β production. Knockout or inhibition of Nox2-derived ROS production protects brain from aging-associated oxidative damage, microglial dysfunction and inflammation.

## Materials and Methods

### Reagents

Amyloid beta peptide (1–42) (Aβ_42_) and scrambled control peptide (SCP) were purchased from Cambridge Biosciences UK and prepared according to the manufacturer’s instruction. The scrambled control peptide (SCP) and Nox2tat ([H]-RKKRRQRRRCSTRVRRQL-[NH2]) were provided by PeptideSynthetics (PPR Ltd. Fareham, UK). A mouse microglial cell line (BV-2) was obtained from Banca Biologica e Cell Factory, Genova, Italy. IL-1β ELISA (mouse and human) kits were from Thermofisher Scientific (UK). DHE (dihydroethidium) was from Invitrogen (UK). Antibodies against phos-p47^phox^ (Ser359), phos-ERK1/2 (Thr202/Tyr204) and 3-nitrotyrosine were from Sigma (UK). Antibodies against Nox2, Iba-1 and Aβ (D-17, sc-5399) were from Santa Cruz Biotechnology (UK). Antibodies against phos-p38MAPK (Thr180/Tyr182) was from Cell Signaling Technology (UK). All other reagents and chemicals were from Sigma unless stated otherwise in the text.

### Animals

The animal studies were performed in accordance with the protocols approved by both the University Ethics Committee and the UK Home Office under the Animals Act (Scientific Procedures) 1986, UK. Nox2KO mice on a C57BL/6J background were originally obtained from Jackson Laboratories (strain B6.129S6-Cybb/J, stock number: 002365), USA. Littermates of WT and Nox2KO mice were bred in our institution from heterozygotes and genotyped. Animals were housed under standard conditions with a 12:12 light dark cycle and food and water were available ad libitum. The midbrain tissues (containing hippocampus and ventral tegmental area regions) from littermates of age-matched WT and Nox2KO male mice at young (3–4 month), and aging (20–22 month) were used (n = 9/per group) for the experiments.

### Human post-mortem midbrain tissues

The post-mortem human midbrain tissues (including hippocampus and the VTA) from individuals (Caucasian ethnical origin) were obtained from the UK Medical Research Council Edinburgh Brain & Tissue Bank. This study had ethical approval from the UK National Health Service Research Ethical Committee (East of Scotland Ethics Service) and the institutional (University of Edinburgh, UK) ethics review board. The inclusive criteria were: adults aged between 25–40 for the young group and age > 60 for aging group. The exclusion criteria were: prior medical history of neurodegenerative disease, adults aged between 41–60 and visible midbrain abnormalities at autopsy. Brain sample were collected between 23–126 hours of post-mortem interval; and grouped randomly according to their ages as young (25–38 years, n = 7:6 males, 1 female); and elderly (61–85 years, n = 8: 6 male and 2 female).

### Cell culture

BV-2 mouse microglial cells were cultured in RPMI-1640 medium supplemented with heat-inactivated FBS (10% v/v), L-glutamine (2 mM), streptomycin (100 µg/mL), and penicillin (50 IU/mL). The day prior to treatment, cells were detached by trypsin-EDTA solution, counted and seeded at 1 × 10^5^/mL medium in 35 mm cell culture dishes. Next day, cells were stimulated with a scrambled control peptide (SCP) or with Aβ_42_ at the same concentration (0.1–10 µM) dissolved in PBS for the periods indicated in the figures. For the experiments to detect Nox2 upregulation, activation of signalling pathways and IL-1β production, cells were stimulated for 24 h as described previously^26,27^. PMA (100 ng/ml) and TNFα (100 U/ml) were used (24 h) to activate Nox2 enzyme in some experiments. Cells were then detached and counted. Cell pellets were used for further experiments.

### Cell viability and cytotoxicity assays

Cell viability was examined using CellTiter 96 AQ_ueous_ One Solution Cell Proliferation Assay (MTS) (Promega). Briefly, BV2 cells were seeded in 96-well flat-bottom plates at a density of 1 × 10^4^ cells/well the day before experiment. Next day the medium was replaced with 100 µl of 5% FCS medium containing either SCP or Aβ_42_ at different concentrations (0–10 µM) with or without Nox2tat and incubated for 24 h. The MTS assay reagents were then added according to manufacturer’s instruction and the plates were incubated for 1 h. The absorbances at 490 nm were recorded using a plate reader (Molecular Devices, UK).

The Aβ_42_ toxicity were examined using MTT assay kit (Sigma, UK) according to manufacturer’s instruction. Cells were seeded and stimulated with SCP or Aβ_42_ as described above in phenol red-free culture medium. In some experiments, TNFα was used with or without tiron for 24 h proliferation. The medium was then replaced by 100 µL MTT solution (0.5 mg/mL in phenol red free medium). Following 2 h of incubation, the MTT solution was removed and the crystals formed were dissolved with 100 µL of DMSO and the absorbance was read at wavelength of 569 nm using a plate reader (Molecular Devices).

### ROS measurement

The O_2_^−^ production by living BV2 cells (5 × 10^4^ cells in suspension) was measured by lucigenin (5 μM)-chemiluminescence or by DHE (2 μM) fluorescence for adherent cells cultured onto chamber slides as described previously^18^. The specificity of O_2_^.−^ detection was confirmed by adding tiron (10 mM), a nonenzymatic O_2_^.−^ scavenger or polyethylene glycol-adsorbed–superoxide dismutase (PEG-SOD, 100 U/ml), a free-radical scavenger. NADPH oxidase inhibitor: apocynin (20 μM) or Nox2tat (10 µM) or a flavo-proteins inhibitor, DPI (diphenyleneiodonium, 20 μM) were used to inhibit Nox2 activity. Some experiments were performed using PMA (100 ng/ml) or TNF α (100 U/ml) to activate microglial NADPH oxidase. DHE images were captured digitally and acquired using a ZEISS fluorescence microscope (Axio Scope.A1). The fluorescence intensity was quantified from at least 5 random fields (269.7 × 269.2 µm) per section with 3 sections/sample.

### Immunofluorescence microscopy

The experiments were performed exactly as described previously^18^. Primary antibodies were used at 1:250 dilution. BSA (2%) was used in the place of primary antibodies as a negative control. Biotin-conjugated anti-rabbit or anti-goat IgG (1:1000 dilution) were used as secondary antibodies. Specific binding of antibodies was detected by extravidin-FITC or streptavidin-Cy3. Images were acquired with a ZEISS fluorescence microscope (Axio Scope.A1). Fluorescence intensities were quantified as described above.

### Immunoblotting

This was performed exactly as described previously^18^. The images were captured digitally using an ImageQuant LAS 4000 mini imaging system (GE Healthcare, UK), and the optical densities of the protein bands were normalized to the loading control bands and quantified.

### Detection of brain tissue IL-1β and MDA assay

The brain tissue IL-1β levels were measured in midbrain tissue homogenates using IL-1β ELISA kits (ThermoFisher, UK) according to manufacturer’s instruction. Lipid peroxidation in brain tissue homogenates was detected using MDA assay kit (Sigma-Aldrich, UK) according to manufacturer’s instruction.

### Statistical analysis

Statistical analysis was performed using one-way analysis of variance (ANOVA) followed by Bonferroni post-hoc tests. For experiments using cultured BV2 cells, the assays were performed in duplicates and repeated for 3–5 time using independent cell cultures. The animal data were obtained from 9 mice/group. For human experiments, 7–8 individual midbrain tissues were used per group. Data were expressed as mean ± SD except where specified in the figure legends. P < 0.05 was considered statistically significant.

## Supplementary information

Supplementary Information.
