# Author Correction: Nox2 dependent redox-regulation of microglial response to amyloid-β stimulation and microgliosis in aging

**DOI:** 10.1038/s41598-023-31194-7

**Published:** 2023-03-31

**Authors:** Li Geng, Lampson M. Fan, Fangfei Liu, Colin Smith, Jian-Mei Li

**Affiliations:** 1 School of Biological Sciences, University of Reading, Reading, UK; 2 Faculty of Health and Medical Sciences, University of Surrey, Guildford, UK; 3 Faculty of Cardiovascular Medicine, University of Oxford, Oxford, UK; 4 Centre for Clinical Brain Sciences, University of Edinburgh, Edinburgh, UK

Correction to: *Scientific Reports* https://doi.org/10.1038/s41598-020-58422-8, published online 31 January 2020

The original version of this Article contained an error in Figure 3, panel A, where the P-p47^phox^ immunoblot was inadvertently duplicated from the Iba-1 immunoblot. The original Figure 3 and accompanying legend appear below.

**Figure 3:**
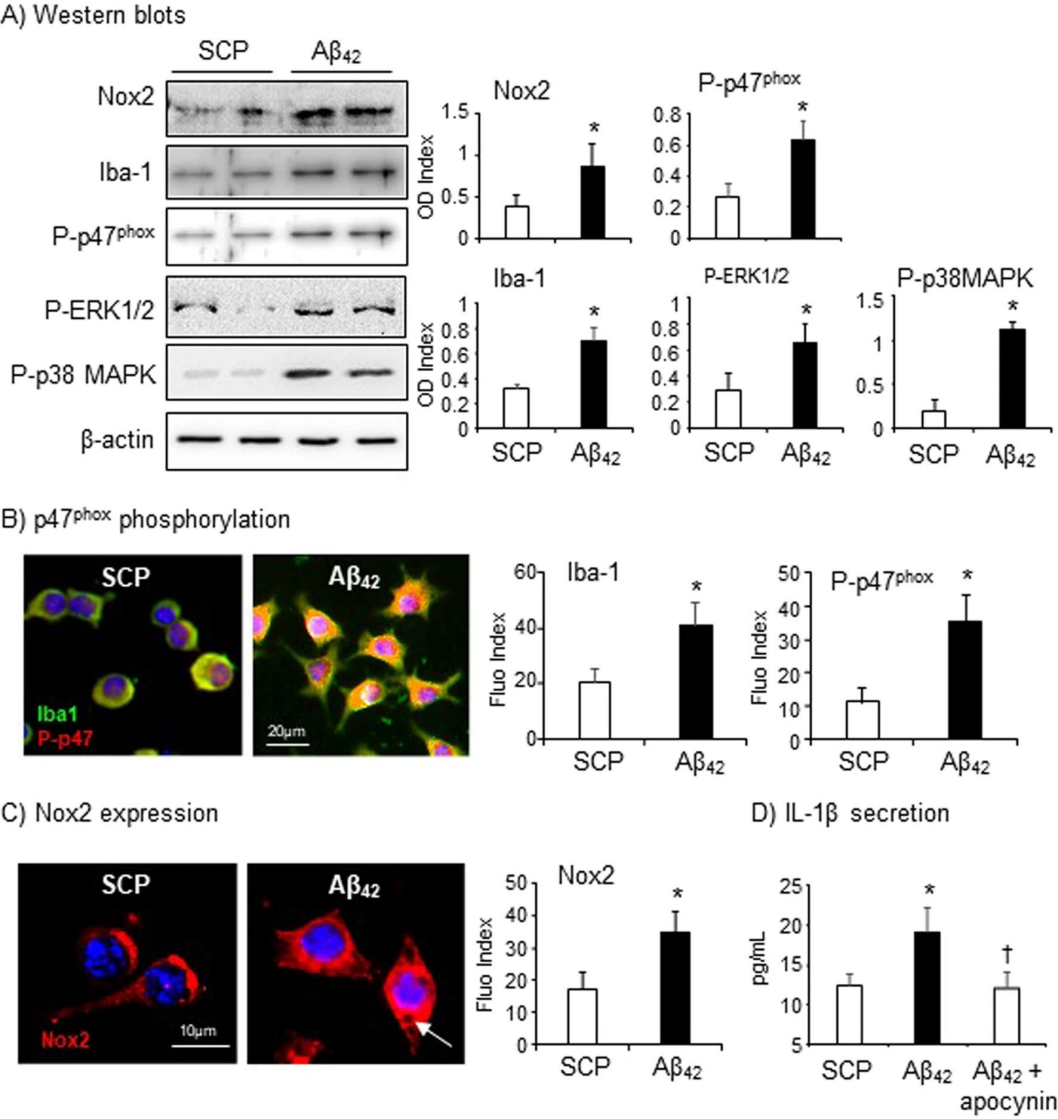
Aβ_42_-induced Iba-1 and Nox2 expression, the activation of stress-signalling pathways and IL-1β secretion by BV2 cells. (**A**) Western blots. Optical densities (ODs) of protein bands were quantified and normalized to β-actin (loading control) detected in the same sample. (**B**) p47^phox^ phosphorylation (red) was detected using a phosphorylation specific antibody against p47^phox^ (Ser359) and double stained with antibody against Iba-1 (green) by immunofluorescence. (**C**) Nox2 expression (red) detected by immunofluorescence. Nuclei were labelled by DAPI (blue) to visualise the cells. Fluorescence intensities were quantified, and expressed as index against controls without primary antibody. (**D**) IL-1β detected in the culture media by ELISA. n = 5 independent cell cultures. *P < 0.05 for indicated values versus SCP values. ^†^P < 0.05 for indicated values versus Aβ_42_ values.

Furthermore, the Supplementary Information file published with this Article contained an error, where the raw data of the Phos-p47^phox^ immunoblot was an inadvertent duplication of the Iba-1 raw data immunoblot (both shown in Figure 3, panel A). In addition, the raw data for Figure 4, panel C was omitted from the Supplementary Information file. The original Supplementary Information file is provided below.

The original Article and accompanying Supplementary Information file have been corrected.

## Supplementary Information

Supplementary Information.

