# Supplementary material for: Nox2 dependent redox-regulation of microglial response to amyloid-β stimulation and microgliosis in aging
Source: Sci Rep. 2020 Jan 31;10:1582. doi: 10.1038/s41598-020-58422-8 (PMC6994719; doi:10.1038/s41598-020-58422-8)

Supplemental information

Nox2 dependent redox-regulation of microglial response to amyloid-β stimulation and microgliosis in aging

Li Geng[1,2,] Lampson M Fan[3], Fangfei Liu[1], Colin Smith[4] and Jian-Mei Li[1,2]

School of Biological Sciences, University of Reading, UK[1]; Faculty of Health and Medical Sciences, University of Surrey, UK[2]; Faculty of Cardiovascular Medicine, University of Oxford, UK[3]; Centre for Clinical Brain Sciences, University of Edinburgh, Edinburgh, UK[4]

Full unedited gels of Western blots in Figure 3A

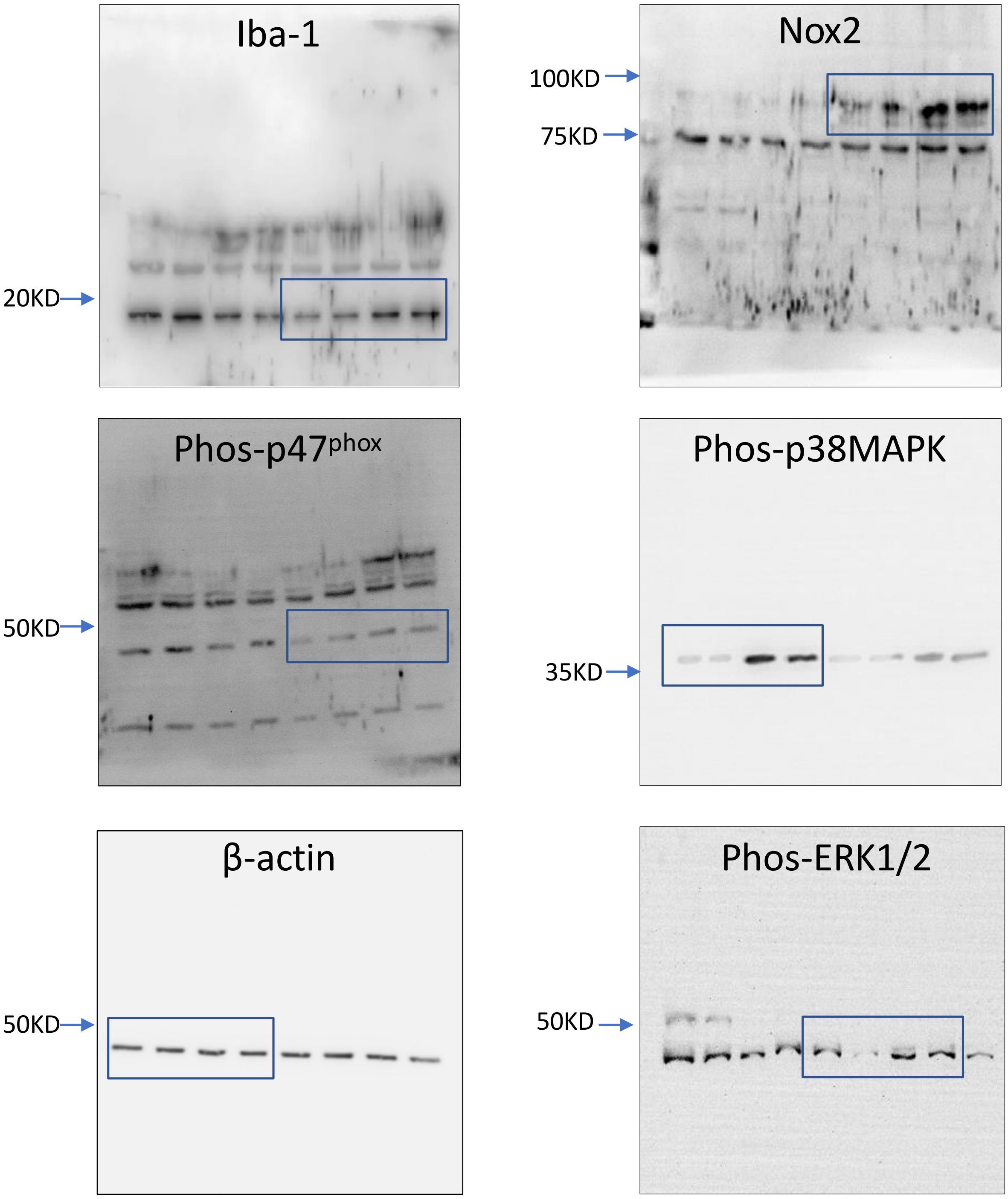

Full unedited gels of Western blots in Figure 3A

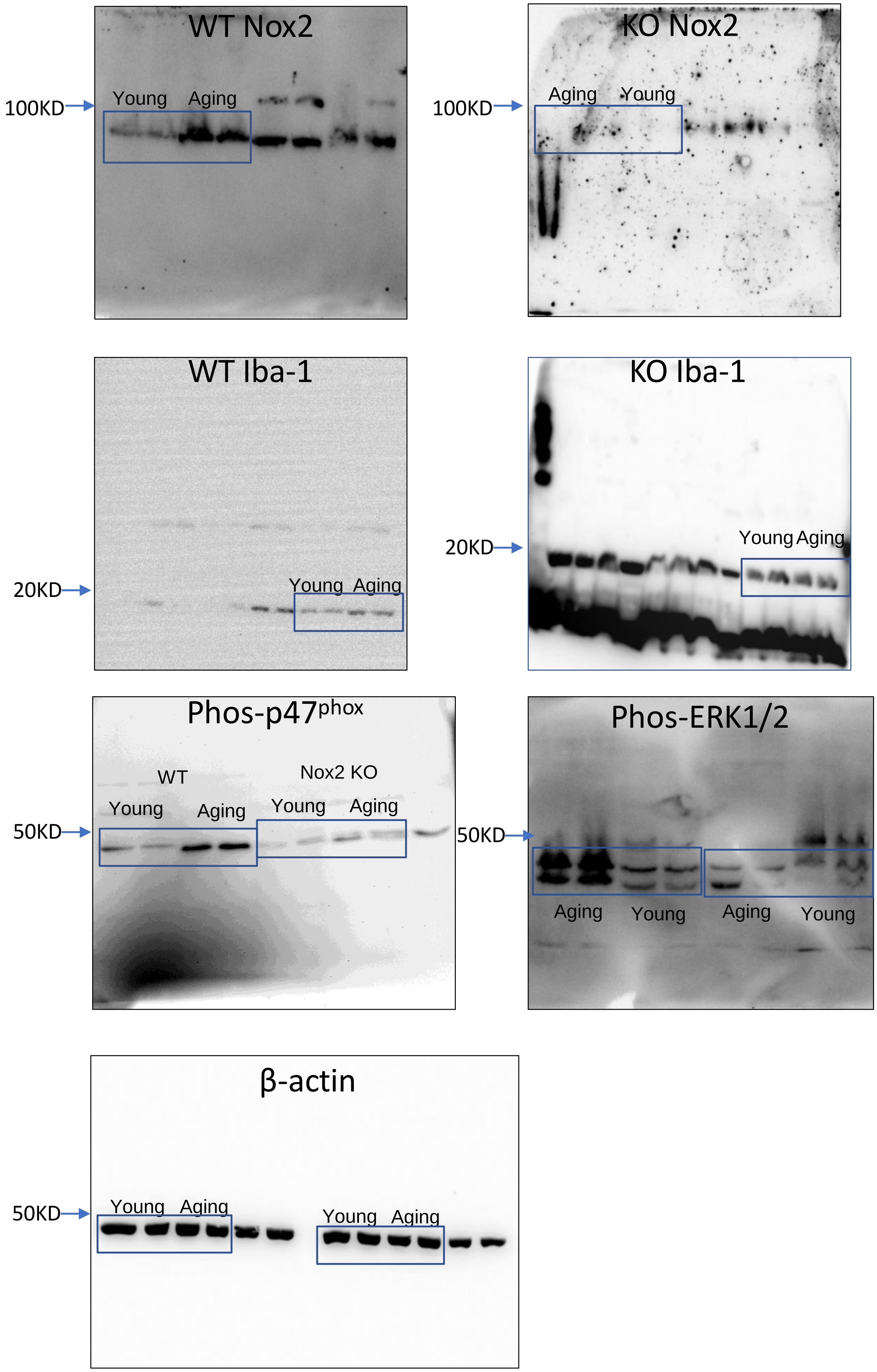

Supplement: Supplementary file 1 — Supplementary Information. [file 41598_2020_58422_MOESM1_ESM.pdf]
