# Supplementary material for: Author Correction: Nox2 dependent redox-regulation of microglial response to amyloid-β stimulation and microgliosis in aging
Source: Sci Rep. 2023 Mar 31;13:5294. doi: 10.1038/s41598-023-31194-7 (PMC10066298; doi:10.1038/s41598-023-31194-7)

Supplemental information

Nox2 dependent redox-regulation of microglial response to amyloid-β stimulation and microgliosis in aging

Li Geng[1,2,] Lampson M Fan[3], Fangfei Liu[1], Colin Smith[4] and Jian-Mei Li[1,2]

School of Biological Sciences, University of Reading, UK[1]; Faculty of Health and Medical Sciences, University of Surrey, UK[2]; Faculty of Cardiovascular Medicine, University of Oxford, UK[3]; Centre for Clinical Brain Sciences, University of Edinburgh, Edinburgh, UK[4]

Full unedited gels of Western blots in Figure 3A

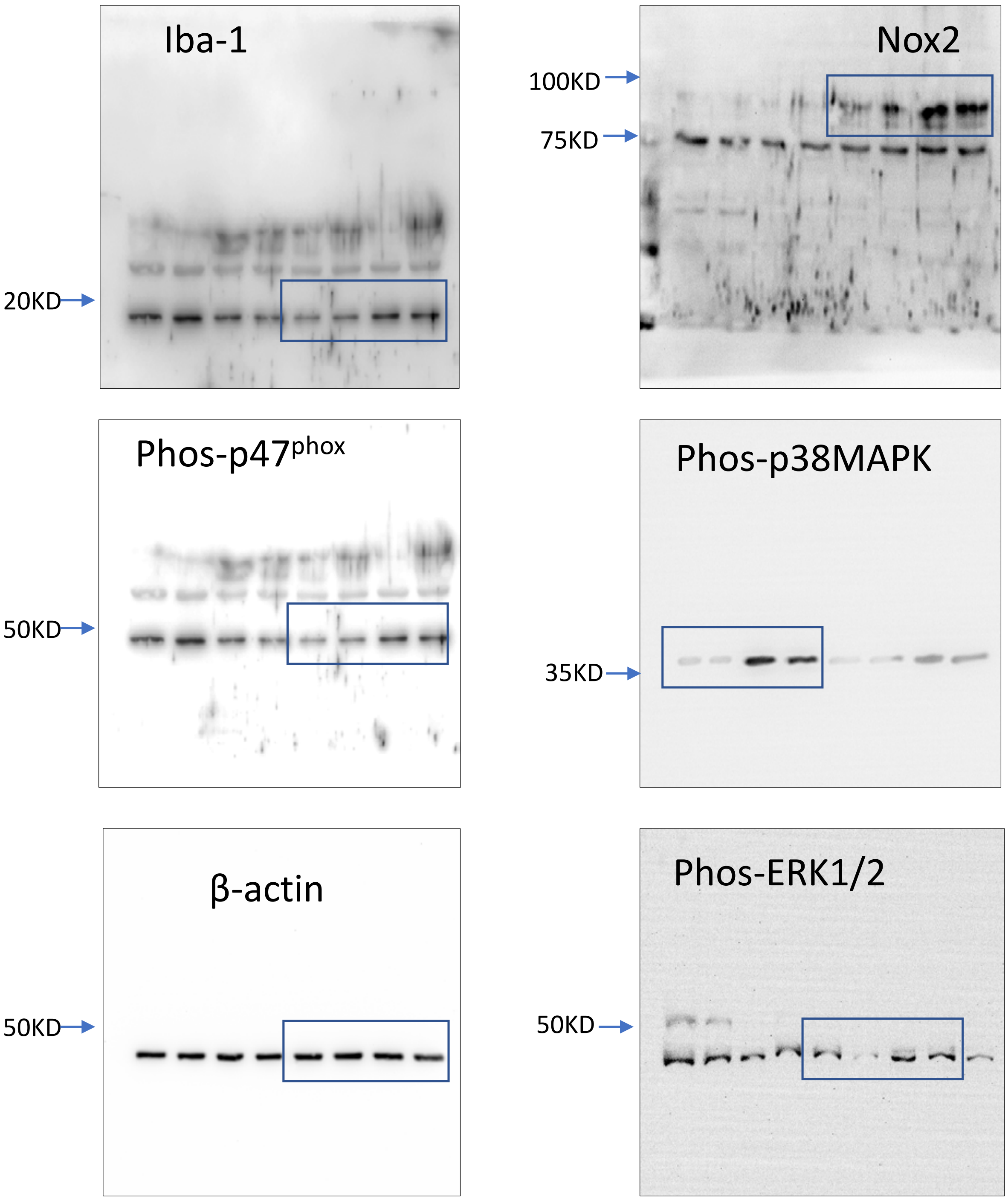

Supplement: Supplementary file 1 — Supplementary Information. [file 41598_2023_31194_MOESM1_ESM.pdf]
